# Association of anaesthesia technique with 30-day primary graft patency after open lower limb revascularization: retrospective cohort study

**DOI:** 10.1093/bjsopen/zrac061

**Published:** 2022-06-03

**Authors:** Janny Xue Chen Ke, Alana M. Flexman, Stephan K. W. Schwarz, Shaun MacDonald, Christopher Prabhakar

**Affiliations:** Department of Anesthesia, St. Paul’s Hospital, Providence Health Care, Vancouver, British Columbia, Canada; Department of Anesthesiology, Pharmacology & Therapeutics, University of British Columbia, Vancouver, British Columbia, Canada; Department of Anesthesiology, Pain Management, and Perioperative Medicine, Dalhousie University, Halifax, Nova Scotia, Canada; Department of Anesthesia, St. Paul’s Hospital, Providence Health Care, Vancouver, British Columbia, Canada; Department of Anesthesiology, Pharmacology & Therapeutics, University of British Columbia, Vancouver, British Columbia, Canada; Department of Anesthesia, St. Paul’s Hospital, Providence Health Care, Vancouver, British Columbia, Canada; Department of Anesthesiology, Pharmacology & Therapeutics, University of British Columbia, Vancouver, British Columbia, Canada; Division of Vascular Surgery, St. Paul’s Hospital, The University of British Columbia, Vancouver, British Columbia, Canada; Department of Anesthesia, St. Paul’s Hospital, Providence Health Care, Vancouver, British Columbia, Canada; Department of Anesthesiology, Pharmacology & Therapeutics, University of British Columbia, Vancouver, British Columbia, Canada

## Abstract

**Background:**

The relationship between anaesthetic technique and graft patency after open lower limb revascularization is unclear. The aim of this study was to evaluate the association between 30-day graft patency after elective infrainguinal bypass and anaesthetic technique (regional anaesthesia (RA, i.e. neuraxial and/or peripheral nerve blockade) compared with general anaesthesia (GA)).

**Methods:**

Patients who underwent elective infrainguinal bypass in the 2014–2019 National Surgical Quality Improvement Program Vascular Procedure Targeted Lower Extremity Open data set were included. Excluded patients were those under 18 years old, those who did not receive RA or GA, and/or had an international normalized ratio of 1.5 of greater, a partial thromboplastin time more than 35 s, or a platelet count less than 80 × 10^9^/L. The primary outcome was primary graft patency without reintervention. The relationship between anaesthetic technique and patency was analysed with multivariable logistic regression.

**Results:**

Included were 8893 patients with a mean(s.d.) age of 68(11) years and 31.5 per cent female. Within the cohort, 7.7 per cent (*n* = 688) patients received RA only, 90.4 per cent (*n* = 8039) GA only, and 1.9 per cent (*n* = 166) both GA and RA. In the RA-only group, 91.7 per cent (631 of 688) received neuraxial anaesthesia. The primary patency rate was 93.2 per cent (573 of 615) for RA only, and 91.5 per cent (6390 of 6983) for GA only (standardized mean difference, 0.063). RA was not associated with a higher rate of patency compared with GA (adjusted OR, 1.16; 95 per cent c.i., 0.83 to 1.63; *P* = 0.378).

**Conclusion:**

There was no association between anaesthetic technique and 30-day graft patency after elective infrainguinal bypass surgery. Further prospective studies would be useful to study the impact of anaesthesia technique on important patient-centred outcomes such as long-term patency and non-home discharge.

## Teaser

Graft patency after revascularization is associated with higher quality of life scores and is an important patient-centred outcome. We evaluated the association between 30-day graft patency after elective infrainguinal bypass surgery and anaesthetic technique (regional anaesthesia (neuraxial and/or peripheral nerve blockade) compared with general anaesthesia). Multivariable logistic regression did not show an association between anaesthetic technique and 30-day graft patency.

## Introduction

Lower limb (infrainguinal) revascularization surgeries are performed for patients with arterial occlusion, with the goals of improving pain and function^1,2^. Graft patency is associated with higher quality of life scores^3^; however, open lower limb revascularization is associated with a significant risk of graft failure, with 30-day patency rates ranging from 78.6 per cent for patients with acute limb ischaemia^4^ to 92.7 per cent in non-emergent revascularizations^5^. Patients with loss of graft patency may require further surgeries and/or amputation^2^. A regional multicentre retrospective cohort of 2036 infrainguinal bypass surgeries from 2003 to 2007 reported a 1-year permanent graft occlusion rate of 12 per cent, and 42 per cent of these patients required major amputation^6^.

Multiple anaesthesia options exist for elective infrainguinal bypass, including general anaesthesia (GA) and regional anaesthesia (RA). RA includes spinal, epidural, and combined spinal epidural anaesthesia as well as peripheral nerve blockade^7^. Rarely, a patient may receive both GA and RA, such as conversion to GA after failed RA. Clinically, major determinations for the choice between RA or GA include contraindications to regional anaesthesia (such as coagulopathy), patient preference, patient factors leading to a higher risk of complications with GA, and surgical factors including duration, complexity, and need for vein harvest from an upper extremity.

As increasingly complex patients undergo infrainguinal revascularization^8^, further additional evidence is required to define the relationship between anaesthetic technique and graft patency, which represents a potentially modifiable factor. Early studies suggested a possible association between neuraxial anaesthesia and decreased rates of reoperation^9,10^ and graft failure^10,11^, but subsequent studies (using data from before 2011) did not find an association between anaesthesia technique and 7-day^12^ or 30-day graft failure^5^. Plausible mechanisms by which RA may impact graft patency include sympathetic blockade, decreased catecholamine release, and improved lower limb blood flow^5^. In the context of arteriovenous fistula creation, regional anaesthesia is associated with improved graft patency compared with general^13^ or local anaesthesia^14^.

The primary objective of this study was to determine the association between anaesthetic technique (RA *versus* GA) and 30-day primary graft patency in patients undergoing elective infrainguinal bypass surgery. Secondary, aims were to analyse the relationship between anaesthetic technique and the rates of major reintervention, amputation, bleeding requiring transfusion, or secondary procedure, pneumonia, composite thromboembolism (venous thromboembolism (VTE), myocardial infarction (MI) or stroke), mortality, composite morbidity and mortality, and non-home discharge.

## Methods

With approval from the University of British Columbia Clinical Research Ethics Board (25 January 2021, H20-03437) and a waiver of informed consent, a retrospective cohort study was performed using the National Surgical Quality Improvement Program (NSQIP^®^) data set. The NSQIP general data set was linked with the ‘Vascular Procedure Targeted Datasets Lower Extremity Open (LEO)’. The NSQIP data set is a large, multicentre data set with prospectively collected variables up to 30 days after surgery^15^. The study was registered before undertaking the analysis (registration number: NCT04730310 (http://www.clinicaltrials.gov); 29 January 2021).

### Study population

All patients aged 18 years and older undergoing elective lower extremity open revascularization cases within the NSQIP^®^ LEO data set between 2014 and 2019 were included. Exclusion criteria were patients with ASA physical status V (defined as ‘5-Moribund’), anaesthesia other than GA or RA (‘unknown’, ‘other’, or missing data for both principal and additional anaesthesia techniques), non-elective surgery status, missing procedure type, or those who did not receive a graft (only femoral endarterectomy or profundaplasty). Patients with a platelet count less than 80 × 10^9^/L within 90 days before surgery^16^, international normalized ratio (INR) of 1.5 or higher, or partial thromboplastin time (PTT) greater than 35 s were excluded as they would likely be considered ineligible for RA based on guidelines^17,18^.

### Anaesthetic technique

The exposure to either RA or GA was modelled as a binary variable in the primary analysis (*Appendix S1* includes detailed definitions). RA was defined as any of spinal, epidural, and/or peripheral nerve block. In NSQIP, RA with monitored anaesthesia care (MAC) was coded as MAC for the principal technique and was included as RA for the analysis. Those receiving both GA and RA (GA + RA) were excluded from the primary analysis but included in planned sensitivity analysis.

### Primary outcome

All outcomes were measured within 30 days after surgery, except for hospital length of stay (LOS) which was the total LOS^15^. The primary outcome, primary graft patency, was defined by NSQIP as one of the following: most severe procedural outcome; clinically patent graft; patent graft, no stenosis; or patent graft with stenosis. Non-patency was defined by having the ‘most severe procedural outcome’ being death, image-proven graft thrombosis, or clinically evident thrombosis with no planned intervention, major amputation, new bypass in the treated arterial segment, not documented, other, revised graft with stenosis, or revised graft, no current stenosis; or untreated loss of patency being ‘yes’ (not patent and no procedure was performed). In the LEO data set, patency documentation included imaging (CT, angiogram, or duplex ultrasound), physical exam, and surgeon’s diagnosis. In NSQIP, the ‘revised graft’ refers to the outcome of requiring graft revision within 30 days after the primary abstracted procedure, not whether the primary procedure itself consisted of a graft revision. NSQIP does not abstract a surgery as the primary procedure if it is performed due to a complication within 30 days of a previous procedure or the same hospitalization.

### Secondary outcomes

Secondary outcomes included major reintervention, amputation, bleeding requiring transfusion, or secondary procedure, VTE, MI or stroke, pneumonia, postoperative LOS, readmission rate, and death^15^, as well as a derived variable of non-home discharge (*Appendix S1*). Two composite secondary outcomes were created: arterial or venous thromboembolism, and composite morbidity and mortality (bleeding, arterial or venous thromboembolism, pneumonia, or death).

### Potential covariates

In building the multivariable logistic regression model, several potential confounding variables were considered based on both previous literature and baseline differences between the two cohorts: age^5,19,20^, bleeding diathesis^5^, severe chronic obstructive pulmonary disease (COPD)^5,19,20^, smoking status, renal failure^19^, functional status^5,6^, diabetes^20^, total operating time^5,19^, year of surgery, high-risk physiological and anatomical risk factors as defined by NSQIP^15^, and procedure type (*Appendix S1*).

## Statistical analysis

Patient characteristics for preoperative, intraoperative, and postoperative variables in the RA, GA, and GA + RA groups were compared with the use of descriptive statistics. Continuous variables are presented as mean(s.d.) and median (interquartile range (i.q.r.)) for parametric and non-parametric data respectively. Categorical variables are presented as frequency (per cent). Standardized mean difference or Hodges­Lehmann statistic was presented. Utilization of RA between 2014 and 2019 is displayed graphically. Complete case analyses without imputation for missing values were performed.

To assess the association among anaesthesia type and patency, a multivariable logistic regression was performed, adjusting for potential confounders as above. The significance level was set at *P* < 0.05 for all analyses. The same exposure and confounder variables were used in the multivariable logistic regressions for secondary outcomes, except that LOS was modelled by way of multivariable linear regression with logarithmic transformation of LOS due to its non-normal distribution. The degree of unmeasured confounding was assessed by way of the *E* value^21^.

In a planned sensitivity analysis, several subgroups were analysed: neuraxial anaesthesia compared with GA, surgical site (femoral *versus* femoropopliteal *versus* popliteal), and previous intervention using the NSQIP variable ‘high-risk factors—anatomical features’. It was not possible to perform subgroup analyses for patients receiving peripheral nerve blocks due to low frequency and lack of detailed block information within NSQIP. In another *a priori* sensitivity analysis, the GA + RA group was analysed as a distinct category in addition to GA-only or RA-only, as well as by grouping GA + RA into either the GA or RA group. Statistical analyses were performed with SAS 9.4 (SAS Institute, Cary, North Carolina, USA). Standardized differences were calculated with the SAS macro ‘Stddiff’^22^.

### Sample size calculation

Grip and colleagues previously reported a 30-day patency rate of 78.6 per cent (mean (s.d.) 1.8 per cent)^3^, although this estimate was not stratified by anaesthesia type. Based on previous NSQIP infrainguinal revascularization studies^4,6^, a 10 per cent rate of RA utilization was assumed. To detect an increase in patency rate by 4 per cent in the RA group (*versus* GA), with an α of 0.05 and power of 0.9, it was calculated that 11 600 patients were required. Data from 2014 to 2019 were included in an attempt to achieve this sample size.

## Results

### Cohort characteristics

**Fig. 1:**
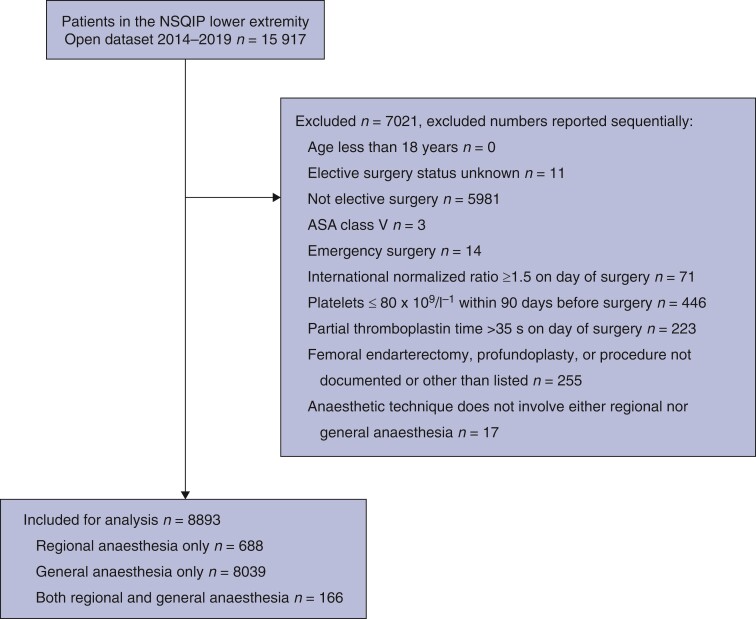
Study inclusion and exclusion flow chart

The cohort included 8893 patients (*Fig. 1*), with a mean(s.d.) age of 68(11) years, and 31.5 per cent (2799 of 8893) were female. Variables with more than 2 per cent missing data were patency (13.0 per cent; *n* = 1155), race (19.3 per cent; *n* = 1713), additional anaesthesia technique (a NSQIP variable for any additional anaesthesia technique that was not considered the primary anaesthesia technique) (90.3 per cent; *n* = 8031), preoperative PTT (46.2 per cent; *n* = 4114), and INR (30.1 per cent; *n* = 2673). The frequencies of missing data were 10.6 per cent (73 of 688) and 13.1 per cent (1056 of 6390) for the RA-only and GA-only groups respectively.

**Fig. 2:**
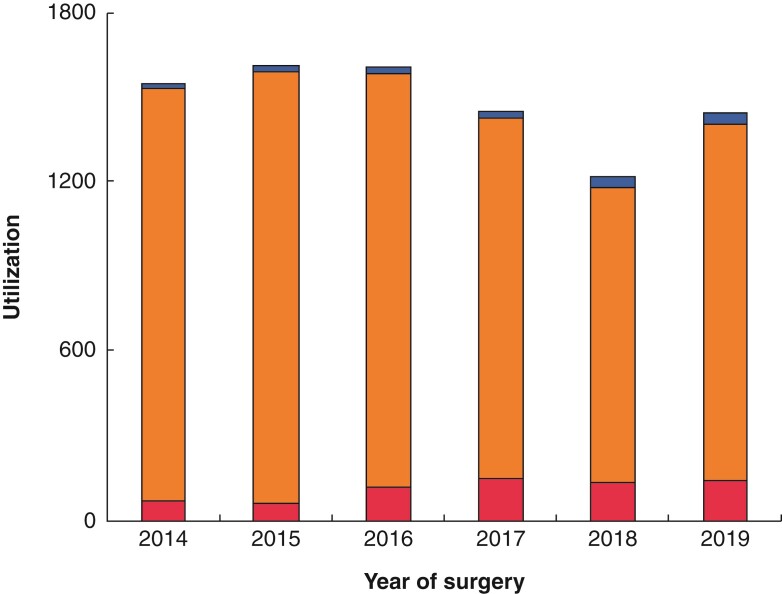
**Regional anaesthesia utilization *versus* year of surgery** The utilization of regional anaesthesia (red, lower section of bar), general anaesthesia (orange, middle section of bar), and both general and regional anaesthesia (blue, top section of bar) are displayed. The percentage of revascularization surgeries performed with regional anaesthesia only between 2014 and 2019 (per year) were 4.7 (72 of 1549), 4.1 (66 of 1613), 7.5 (121 of 1609), 10.2 (148 of 1453), 11.0 (135 of 1224), and 10.1 (146 of 1445) respectively (*P* < 0.0001, Cochran–Armitage trend test, two-sided)

The breakdown of the cohort by anaesthesia technique is 7.7 per cent (*n* = 688) of patients received RA-only, 90.4 per cent (*n* = 8039) received GA-only, and 1.9 per cent (*n* = 166) received both GA and RA. Of those who received any RA, 85.8 per cent (733 of 854) received neuraxial anaesthesia. Of those with RA-only, neuraxial use was 91.7 per cent (631 of 688; *n* = 561 spinal and *n* = 159 epidural anaesthesia), and 4.4 per cent (30 of 688) received ‘regional anaesthesia’. In the GA + RA group, 61.5 per cent (102 of 166) involved neuraxial anaesthesia, with 36.7 per cent (61 of 166) receiving ‘regional anaesthesia’ (definitions in *Appendix S1*). *Fig. 2* shows utilization of RA between 2014 and 2019.

The study population characteristics are summarized in *Table 1*. Compared with patients in the GA-only group, patients in the RA-only group were more likely to have high-risk physiological factors (definition in *Appendix S1*). In contrast, patients receiving GA-only were more likely to have bleeding disorders, previous intervention, longer duration of surgery, and complex grafts. The characteristics of patients in the GA + RA group are presented in *Table S1*.

**Table 1:** Cohort characteristics

Characteristic	Regional only *N* = *688*	General only *N* = 8039	*P*	Standardized difference
**Age (years), mean(s.d.)**	71.2(10.2)	67.2(10.5)	<0.001	0.389
**Female sex**	205 (29.8)	2548 (31.7)	0.304	−0.041
**ASA physical status**			<0.001	0.166
I	0	14 (0.2)		
II	29 (4.2)	363 (4.5)		
III	469 (68.2)	5981 (74.4)		
IV	187 (27.2)	1671 (20.8)		
**Preoperative functional health status**			0.083	0.153
Independent	668 (97.1)	7644 (95.1)		
Partially dependent	18 (2.6)	348 (4.3)		
Totally dependent	0	23 (0.3)		
**Current smoker, within 1 year before surgery**	246 (35.8)	3540 (44.0)	<0.001	−0.170
**History of severe COPD**	102 (14.8)	988 (12.3)	0.054	0.074
**Bleeding disorders and anticoagulants**	29 (4.2)	1579 (19.6)	<0.001	−0.490
**Preoperative antiplatelet medication**	533 (77.5)	6822 (84.9)	<0.001	−0.189
**Platelets (10** ^**9**^ **/l), median (i.q.r.)**	231 (187–284)	235 (191–288)	0.469	2 (−8 to 4)*
**Congestive heart failure, within 30 days before surgery**	7 (1.0)	146 (1.8)	0.126	−0.068
**Hypertension requiring medication**	551 (80.1)	6530 (81.2)	0.463	−0.029
**Preoperative renal failure**	248 (36.1)	2521 (31.4)	0.011	0.099
Acute renal failure within 24 h before surgery	2 (0.3)	49 (0.6)	0.292	−0.048
Currently on dialysis	28 (4.1)	340 (4.2)	0.842	−0.008
**Diabetes**			0.614	0.041
On insulin	151 (22.0)	1879 (23.4)		
On non-insulin medication	131 (19.0)	1563 (19.4)		
**Symptomatology**			0.012	0.131
Asymptomatic	33 (4.8)	299 (3.7)		
Claudication	251 (36.5)	2855 (35.5)		
Critical limb ischaemia: rest pain	148 (21.5)	2190 (27.2)		
Critical limb ischaemia: tissue loss	249 (36.2)	2602 (32.4)		
**Procedure type**			<0.001	0.438
Femoral distal bypass with prosthetic/spliced vein/composite	29 (4.2)	791 (9.8)		
Femoral distal bypass with single segment saphenous vein	155 (22.5)	1489 (18.5)		
Femoropopliteal bypass with prosthetic/spliced vein/composite	122 (17.7)	2281 (28.4)		
Femoropopliteal bypass with single segment saphenous vein	326 (47.4)	2812 (35.0)		
Popliteal distal bypass with prosthetic/spliced vein/composite or non-saphenous conduit	2 (0.3)	155 (1.9)		
Popliteal distal with single segment saphenous vein	54 (7.9)	511 (6.4)		
**High-risk anatomical factors**			<0.001	0.238
Prior ipsilateral bypass involving currently treated segment	95 (13.8)	1568 (19.5)		
Prior ipsilateral percutaneous intervention involving currently treated segment	84 (12.2)	1434 (17.8)		
**High-risk physiological factors**	181 (26.3)	1328 (16.5)	<0.001	0.240
**Total operating time (min), median (i.q.r.)**	168 (132.5–223.5)	210 (154–284)	<0.001	−38 (−44 to −31)*
**Race**			<0.001	0.875
White	270 (39.2)	5401 (67.2)		
Black or African American	39 (5.7)	1281 (15.9)		
Unknown/not reported	366 (53.2)	1280 (15.9)		
Asian, Native Hawaiian, or Pacific Islander, American Indian or Alaska Native	13 (1.9)	77 (1.0)		

* Hodges–Lehmann estimator midpoint (95 per cent c.i.). Numbers are n (%) unless otherwise specified. COPD, chronic obstructive pulmonary disease; i.q.r., interquartile range.

#### Graft patency

After excluding patients with missing patency data, the rate of patent graft for the overall cohort was 91.6 per cent (7086 of 7738). By anaesthetic technique, the patency rate was 93.2 per cent (573 of 615) for RA and 91.5 per cent (6390 of 6983) for GA (standardized mean difference, 0.063). The characteristics of patients with missing data for patency are listed in *Table S2* and differed from the main cohort in terms of more asymptomatic patients and fewer previous bypasses. The patency rates for different procedure types are presented in *Table 2*.

**Table 2:** Patency rates by surgery type

Procedure	*n* (% total cohort) *n* = 8893	Patency (*n*, % row total)		
		Missing *n* = 1155 (13.0)	No *n* = 652 (7.3)	Yes *n* = 7086 (79.7)
**Femoral distal bypass**				
with single segment saphenous vein	1687 (19.0)	182 (10.8)	176 (10.4)	1329 (78.8)
with prosthetic/spliced vein/composite	832 (9.4)	108 (13.0)	105 (12.6)	619 (74.4)
**Femoropopliteal bypass**				
with single segment saphenous vein	3198 (36.0)	455 (14.2)	186 (5.8)	2557 (80.0)
with prosthetic/spliced vein/composite	2449 (27.5)	303 (12.4)	127 (5.2)	2019 (82.4)
**Popliteal distal bypass**				
with single segment saphenous vein	568 (6.4)	83 (14.6)	41 (7.2)	444 (78.2)
with prosthetic/spliced vein/composite or non-saphenous conduit	159 (1.8)	24 (15.1)	17 (10.7)	118 (74.2)

### Outcomes and multivariable regression

The use of RA only compared with GA only, was not associated with a higher rate of patency (adjusted OR, 1.16; 95 per cent c.i., 0.83 to 1.63; *P* = 0.378). The model had a receiver operating characteristic (ROC) area under the curve of 0.645, with a Nagelkerke *R*^2^ of 0.0446.

The ORs of RA compared with GA for the primary and secondary outcomes are listed in *Table 3*, after adjusting for covariates listed above. The ORs for patency comparing RA *versus* GA in subgroups of surgery by location and by history of previous intervention are listed in *Table 4*. Sensitivity analysis using different groupings of RA, GA, and GA + RA did not change the associations.

**Table 3:** Outcomes and logistic regression ORs for primary and secondary outcomes

Outcome	Regional only *N* = 688 Number (%)	General only *N* = 8039 Number (%)	Standardized difference	Adjusted* OR for regional only (95% c.i.)	*P*†
**Patency**	573 (93.2)	6390 (91.5)	0.063	1.16 (0.83 to 1.63)	0.378
**Major reintervention on the bypass**	18 (2.6)	332 (4.1)	−0.084	0.71 (0.43 to 1.16)	0.173
**Major amputation (transtibial/proximal)**	6 (0.9)	132 (1.6)	−0.069	0.60 (0.26 to 1.39)	0.237
**Unplanned readmission**	105 (15.3)	1104 (13.7)	0.043	1.19 (0.95 to 1.50)	0.126
**Non-home discharge**	120 (17.5)	1673 (20.9)	−0.086	0.74 (0.59 to 0.93)	**0**.**009**
**Composite (morbidity and mortality)**	100 (14.5)	1250 (15.6)	−0.028	1.10 (0.87-1.39)	0.429
Death	7 (1.0)	88 (1.1)	−0.008	0.76 (0.34-1.70)	0.505
Bleeding requiring transfusion or procedure	76 (11.1)	1010 (12.6)	−0.047	1.12 (0.0.86-1.46)	0.409
Pneumonia	8 (1.2)	76 (1.0)	0.021	1.34 (0.62 to 2.90)	0.452
Arterial and venous thromboembolism‡	26 (3.8)	267 (3.3)	0.025	1.12 (0.73 to 1.72)	0.605

*Covariates adjusted for were age, bleeding disorder, severe chronic obstructive pulmonary disease, smoking status, renal failure, functional status, diabetes, total operating time, year of surgery, high-risk physiological and anatomical risk factors as defined by National Surgical Quality Improvement Program, and procedure type. †Multivariable logistic regression. ‡Myocardial infarction, stroke, venous thromboembolism.

**Table 4:** Subgroup analysis for patency with multivariable regressions

Subgroup	*N* modelled	Adjusted* OR (95% c.i.) for regional *versus* general	*P*
**Neuraxial anaesthesia with/without general† *versus* general anaesthesia only**	7638	1.08 (0.79–1.48)	0.638
**Neuraxial only *versus* general only**	7546	1.25 (0.88–1.80)	0.218
**Spinal only *versus* general only**	7481	1.25 (0.85–1.83)	0.251
**Femoral bypass**	2179	1.50 (0.84–2.67)	0.172
**Femoropopliteal bypass**	4803	0.92 (0.60–1.41)	0.692
**Popliteal bypass**	616	2.35 (0.54–10.31)	0.258
**Previous bypass‡**	2792	0.96 (0.54–1.72)	0.889

* Covariates adjusted for were age, bleeding disorder, severe chronic obstructive pulmonary disease, smoking status, renal failure, functional status, diabetes, total operating time, year of surgery, high-risk physiological and anatomical risk factors as defined by National Surgical Quality Improvement Program, and procedure type. †Includes patients receiving only neuraxial or both general and neuraxial anaesthesia. ‡Defined as previous ipsilateral bypass or percutaneous intervention involving currently treated segment in National Surgical Quality Improvement Program. Values are *n* (%) unless stated otherwise.

The median (i.q.r.) (range) for LOS was 4 (3–6) (0 to 35) and 4 (3–6) (0 to 88) days for RA only and GA only respectively, with a Hodges–Lehmann statistic of 0.5 days (95 per cent c.i. 0 to 1). Due to the right skewed non-normal distribution, LOS was analysed using log transformed LOS in a multivariable linear regression. The RA-only technique was associated with a 12.5 per cent increase in LOS (95 per cent c.i. 7 to 16 per cent) in the regional group (*P* < 0.0001). Analyses of LOS using a Poisson regression or multivariable linear regression without log transformation produced similar results. Exploratory analysis with inclusion of ASA physical status and sex did not meaningfully alter the association between anaesthesia technique and patency, non-home discharge, or LOS.

## Discussion

There have been conflicting data on the relationship between anaesthesia technique and graft patency. In this analysis of 8893 patients from a multicentre cohort undergoing elective lower limb revascularization, there was no association between anaesthesia technique and 30-day primary graft patency. Although the study cannot exclude a small effect from anaesthetic technique, the results suggest that any association is not large. Additional sensitivity analyses of surgical subtypes, and specific patient groups revealed similar results, further confirming the robustness of the results.

Primary patency rate was used as the primary outcome as it is a patient-centred outcome that is correlated with quality of life^3^. Compared with the literature, the 30-day patency rate in the current cohort was higher than the 78.6 per cent reported in patients with acute limb ischaemia^4^, but similar to the 92.7 per cent reported in non-emergent patients in the 2005–2008 NSQIP dataset^5^. The literature on the association between anaesthesia technique and graft patency is mixed. In a 1993 randomized trial of 100 patients undergoing elective lower extremity vascular surgery, patients who received GA had higher rates of the secondary outcome of reoperation within the 6-month follow-up interval than patients who received epidural anaesthesia^8^. Subsequent 1993^23^ and 1995^11^ retrospective re-analyses of different randomized trials showed conflicting results. A retrospective single-centre study of 822 patients did not find a difference in 7-day graft occlusion rates, which was approximately 10 per cent in both epidural and GA groups^9^. A retrospective analysis of 1995–2003 Veterans Affairs NSQIP data showed that GA, compared with spinal anaesthesia, was associated with an OR of 1.43 (95 per cent c.i. 1.16 to 1.77; *P* = 0.001) for graft failure, although this study did not adjust for important confounders such as surgical acuity and coagulopathy^10^. In contrast, another 2005–2008 NSQIP analysis found no difference in the secondary outcome of patency^5^.

The present study adds to the literature on anaesthesia type and patency by analysing an updated, contemporary cohort of patients using a specialty-focused data set. The multicentre data set has broad generalizability. The LEO data set contains more detailed clinical information that was not present in previous studies, including clear clinical, and imaging definitions of patency in the data abstraction guides. Moreover, it was possible to control for important confounders not included in some previous studies such as duration of surgery, coagulopathy, and high-risk anatomical factors.

The literature has shown mixed results regarding the superiority of RA over GA for morbidity and mortality^1,5,19,20,24^. In terms of secondary outcomes, no association between regional technique and outcomes was found except for decreased non-home discharge and increased LOS; however, the differences were small, and these exploratory results should be interpreted with caution in the context of small sample size and multiple testing^25^. The difference in LOS may be related to the sicker population in the RA group, and the small magnitude of difference aligns with previous literature of small to no differences in LOS^19,20,24^. While RA was associated with decreased odds of non-home discharge, this may be related to the more complex grafts and surgeries in the GA group, though the mechanism remains to be elucidated given the lack of difference amongst rates of patency, reintervention, morbidity, and mortality outcomes in the current cohort. The *E* value of 1.6 for OR and 1.23 for lower 95 per cent c.i. limit suggest that any residual, unadjusted confounder (such as frailty) would have to have a more than small effect to change the association to null. A recent multivariable modelling of 10 145 patients in the Vascular Study Group of New England database found that preoperative predictors of non-home discharge were age, sex, non-White race, tissue loss, cardiac co-morbidity, partial ambulatory deficit, and insulin-dependent diabetes^26^. Whether anaesthesia technique is a potentially modifiable factor in decreasing non-home discharge should be confirmed in future studies.

The present study should be interpreted in the context of several limitations. In many cases it can be difficult to determine the exact RA technique used due to the coding scheme and missing data. Misclassification of anaesthesia technique was possible depending on the chart documentation and the data abstracter. The principal anaesthesia technique may have been coded as GA even if there was additional RA, and MAC/intravenous sedation when this coexists with RA^15^. Furthermore, additional anaesthesia technique, where RA may be included, is not a mandatory variable and had a high rate of missing data (*n* = 8031). Reassuringly, out of 166 patients who had MAC as the principal anaesthesia technique, 139 patients had an additional anaesthesia technique of either neuraxial anaesthesia, or RA. Sensitivity analysis with the additional grouping of the GA + RA group revealed similar results. There was also a relatively high rate of missing patency values (13 per cent), which may have biased the results by excluding more patients with patent vessels who did not seek follow-up imaging. Other limitations include residual confounding by indication, and variables that were not available in the NSQIP data set, such as hospital-specific factors and anatomical details, which may affect RA utilization and outcomes^27^. Similarly, it was not possible to include cardiac valvular disease as a confounder^20^ as it was not available, though this likely did not affect the results given the less than 2 per cent incidence of heart failure in the cohort. It was also not possible to analyse outcomes beyond 30 days after surgery. Last, the study may be underpowered to detect a small difference due to a smaller than anticipated sample size.

For patients undergoing elective open lower limb revascularization, there was no association between the anaesthetic technique (RA *versus* GA) and 30-day primary patency. Nevertheless, this should be interpreted with caution due to potential misclassification of anaesthesia technique within the NSQIP data set, smaller than projected sample size, and possible residual confounding. Further prospective studies would be useful to study the impact of anaesthesia technique on important patient-centred outcomes such as long-term patency and non-home discharge.

## Supplementary Material

zrac061_Supplementary_Data

## Data Availability

The NSQIP data set used in this study is available to participating sites upon appropriate institutional approval.
