# Supplementary material for: Association of anaesthesia technique with 30-day primary graft patency after open lower limb revascularization: retrospective cohort study
Source: BJS Open. 2022 Jun 3;6(3):zrac061. doi: 10.1093/bjsopen/zrac061 (PMC9164863; doi:10.1093/bjsopen/zrac061)
Supplement: zrac061_Supplementary_Data [file zrac061_supplementary_data.zip › Supplementary_material.docx]

**Supplementary material**

**Appendix S1: Definitions for exposure, outcome, and confounder variables**

Please refer to the NSQIP Participant User Files (particularly Chapter 4, Variables and Definitions) for detailed definitions and data abstraction procedures (15).

1. **Exposure**

The exposure to either regional anesthesia (RA) or general anesthesia (GA) was modelled as a binary variable. RA was defined as any of spinal, epidural, and/or peripheral nerve block (PNB). This was done using NSQIP variables Principal (ANESTHES) or Additional (ANESTHES_OTHER) anesthesia technique character variables containing the terms “spinal”, “epidural”, “regional”, or monitored anesthetic care (MAC). In NSQIP, any regional anesthesia with MAC would be coded as MAC for the principal technique. GA was defined using ANESTHES or ANESTHES_other containing “general”. If both GA and RA were present in the principal and additional anesthesia techniques, this group was labelled as “GA+RA”. The GA+RA group was excluded from the primary analysis, but included in a sensitivity analysis.

1. **Outcomes**

All outcomes were measured within 30 days postoperatively in NSQIP. Primary patency was derived using the NSQIP® Lower Extremity Open (LEO) variables “Most Severe Procedural Outcome” LEO_MOSTSEVOUTCOME and “Untreated Loss of Patency” LEO_ULP. A patent graft was defined as “most severe procedural outcome” being “clinically patent graft”, “patent graft, no stenosis”, or “patent graft with stenosis”. Non-patency was defined by “most severe procedural outcome” being “death”, “image-proven graft thrombosis or clinically evident thrombosis with no planned intervention”, “major amputation”, “new bypass in the treated arterial segment”, “not documented”, “other”, “revised graft with stenosis”, or “revised graft, no current stenosis”; or untreated loss of patency being “yes” (i.e. not patent and no procedure was done). In the LEO dataset, patency documentation included imaging (computerized tomography, angiogram, duplex ultrasound), physical exam, and surgeon’s diagnosis. In NSQIP, the “revised graft” refers to the outcome of requiring graft revision within 30 days after the primary abstracted procedure, not whether the primary procedure itself consisted of a graft revision. NSQIP does not abstract a surgery as the primary procedure if it is performed due to a complication within 30 days of a previous procedure or the same hospitalization.

Secondary outcomes were NSQIP LEO variables major reintervention, amputation, bleeding requiring transfusion or secondary procedure, venous thromboembolism (VTE), myocardial infarction (MI) or stroke, and pneumonia, as well as derived variables non-home discharge, postoperative length of stay, readmission rate, and death. We created two composite secondary outcomes: arterial or venous thromboembolism (AVTE), and composite morbidity and mortality (bleeding, AVTE, pneumonia, or death). Non-home discharge was defined using the NSQIP variable “Discharge Destination”, with yes being “hospice”, “Multi-level Senior Community”, “rehabilitation”, “Separate Acute Care”, “Skilled Care, Not Home”, “Unskilled Facility Not Home”, and no being “home”, “Facility Which was Home”, or “Against Medical Advice”.

1. **Confounders**

Multivariable logistic regression was performed adjusting for the following potential confounders that are collected in NSQIP, based on literature and differences in cohort characteristics: age (5,19,20), bleeding disorders (5), severe chronic obstructive pulmonary disease (COPD) (5,19,20), smoking status, renal failure (19), functional status (5,6), diabetes (20), total operating time (5,19), year of surgery, high risk physiologic and anatomic risk factors as defined by NSQIP, and procedure type.

A patient was considered to have pre-existing renal failure if positive for either NSQIP variables “Acute Renal Failure” OR “Dialysis”, OR GFR ≤ 60mL/min/1.73m^2^ as calculated by the CKD-EPI formula (28) using NSQIP® preoperative creatinine, age, and sex. Within NSQIP, the variable of bleeding disorder is specifically defined as “any chronic/persistent/active condition that places the patient at risk for excessive bleeding (e.g., vitamin K deficiency, hemophilia, thrombocytopenia, chronic anticoagulation therapy that has not been discontinued prior to surgery).” The NSQIP variable of high risk physiologic factors include End Stage Renal Disease (ESRD), age >80, New York Heart Association (NYHA) Congestive Heart Failure (CHF) Class III/IV, Left Ventricular Ejection Fraction (LVEF) < 30%, or unstable angina or myocardial infarction within 30 days preoperatively. Due to NSQIP de-identification where patients aged >90 years were given a value of 90 for age, we recoded age “90+” as 90 to analyse age as a continuous variable.

**Table S1: Cohort characteristics for patients receiving both general and regional anaesthesia.**

| **Variable** | **Regional (N, %)** | **General (N, %)** | **Both (N, %)** |
| --- | --- | --- | --- |
| Total *N* = 8893 | 688 (7.7%) | 8039 (90.4%) | 166 (1.9%) |
| **Preoperative** | | | |
| **Age** | 71.2 (10.2) | 67.2 (10.5) | 67.3 (10.9) |
| **Female sex** | 205 (29.8%) | 2548 (31.7%) | 46 (27.7%) |
| **Race** |  |  |  |
| White | 270 (39.2%) | 5401 (67.2%) | 64 (38.6%) |
| Black or African American | 39 (5.7%) | 1281 (15.9%) | 34 (20.5%) |
| Unknown/not reported | 366 (53.2%) | 1280 (15.9%) | 67 (40.4%) |
| Asian, Native Hawaiian or Pacific Islander, American Indian or Alaska Native | 13 (1.9%) | 77 (1.0%) | 1 (0.6%) |
| **ASA** |  |  |  |
| I | 0 | 14 (0.2%) | 1 (0.6%) |
| II | 29 (4.2%) | 363 (4.5%) | 5 (3.0%) |
| III | 469 (68.2%) | 5981 (74.4%) | 121 (72.9%) |
| IV | 187 (27.2%) | 1671 (20.8%) | 39 (23.5%) |
| Missing | 3 (0.4%) | 10 (0.1%) | 0 |
| **Diabetes on insulin** | 151 (22.0%) | 1879 (23.4%) | 45 (27.1%) |
| **Diabetes on non-insulin medication** | 131 (19.0%) | 1563 (19.4%) | 21 (12.7%) |
| **Current smoker within 1 year** | 246 (35.8%) | 3540 (44.0%) | 76 (45.8%) |
| **Functional health status**  **Prior to Surgery** |  |  |  |
| Independent | 668 (97.1%) | 7644 (95.1%) | 160 (96.4%) |
| Partially Dependent | 18 (2.6%) | 348 (4.35) | 4 (2.4%) |
| Totally Dependent | 0 | 23 (0.3%) | 1 (0.6%) |
| **History of severe COPD** | 102 (14.8%) | 988 (12.3%) | 22 (13.3%) |
| **Congestive heart failure (CHF) in 30 days before surgery** | 7 (1.0%) | 146 (1.8%) | 2 (1.2%) |
| **Hypertension requiring medication** | 551 (80.1%) | 6530 (81.2%) | 137 (82.5%) |
| **Acute renal failure within 24 hours before surgery** | 2 (0.3%) | 49 (0.6%) | 1 (0.6%) |
| **Currently on dialysis (pre-op)** | 28 (4.1%) | 340 (4.2%) | 4 (2.4%) |
| **Renal failure preoperatively (derived)** | 248 (36.05%) | 2521 (31.4%) | 47 (28.3%) |
| **Bleeding disorders and anticoagulants** | 29 (4.2%) | 1579 (19.6%) | 26 (15.7%) |
| **Pre-procedural**  **Antiplatelet Medication** | 533 (77.5%) | 6822 (84.9%) | 136 (81.9%) |
| **Pre-procedural**  **Medication-Statin** | 509 (74.0%) | 5940 (73.9%) | 125 (75.3%) |
| **Platelet (k/mm³)** 10^9^/L (median/IQR) | 231 (187-284) | 235 (191-288) | 243 (196-294) |
| **Symptomatology** | | | |
| Asymptomatic | 33 (4.8%) | 299 (3.7%) | 5 (3.0%) |
| Claudication | 251 (36.5%) | 2855 (35.5%) | 63 (38.0%) |
| Critical limb ischemia: rest pain | 148 (21.5%) | 2190 (27.2%) | 49 (29.5%) |
| Critical limb ischemia: tissue loss | 249 (36.2%) | 2602 (32.4%) | 46 (27.7%) |
| Missing | 7 (1.0%) | 93 (1.2%) | 3 (1.8%) |
| **Intraoperative** | | | |
| **Procedure type** | | | |
| Femoral distal bypass with prosthetic/spliced vein/composite | 29 (4.2%) | 791 (9.8%) | 12 (7.2%) |
| Femoral distal bypass with single segment saphenous vein | 155 (22.5%) | 1489 (18.5%) | 43 (25.9%) |
| Femoropopliteal bypass with prosthetic/spliced vein/composite | 122 (17.7%) | 2281 (28.4%) | 46 (27.7%) |
| Femoropopliteal bypass with single segment saphenous vein | 326 (47.4%) | 2812 (35.0%) | 60 (36.1%) |
| Popliteal distal bypass with prosthetic/spliced vein/composite or non-saphenous conduit | 2 (0.3%) | 155 (1.9%) | 2 (1.2%) |
| Popliteal distal with single segment saphenous vein | 54 (7.9%) | 511 (6.4%) | 3 (1.8%) |
| **High Risk Factors, Anatomic** | | | |
| Prior ipsilateral bypass involving currently treated segment | 95 (13.8%) | 1568 (19.5%) | 26 (15.7%) |
| Prior ipsilateral percutaneous intervention involving currently treated segment | 84 (12.2%) | 1434 (17.8%) | 33 (19.9%) |
| **Total operating time** (minutes)  (median/IQR) | 168 (132.5-223.5) | 210 (154-284) | 225.5 (183-293) |
| **High**  **Risk Factors**, Physiologic | 181 (26.3%) | 1328 (16.5%) | 35 (21.1%) |
| **Postoperative** | | | |
| **Patency % when excluding patients with missing values** | 573 (93.2%) | 6390 (91.5%) | 123 (87.9%) |
| **Patency % when including patients with missing values (missing *N* =1155)** | 573 (82.3%) | 6390 (79.5%) | 123 (74.1%) |
| **Patency** | 73 (10.6%) | 1056 (13.1%) | 26 (15.7%) |
| 'Clinically Patent Graft' | 506 (73.6%) | 5230 (65.1%) | 98 (59.0%) |
| 'Patent graft with stenosis' | 8 (1.2%) | 135 (1.7%) | 6 (3.6%) |
| 'Patent graft, no stenosis' | 59 (8.6%) | 1025 (12.8%) | 19 (11.5%) |
| Untreated  Loss of Patency | 12 (1.7%) | 116 (1.4%) | 3 (1.8%) |
| **Major reintervention**  **on the bypass** | 18 (2.6%) | 332 (4.1%) | 9 (5.4%) |
| **Major**  **Amputation (Transtibial**  **or Proximal)** | 6 (0.9%) | 132 (1.6%) | 3 (1.8%) |
| **Unplanned readmission**  **[based on readmission 1]** | 105 (15.3%) | 1104 (13.7%) | 26 (15.7%) |
| **Postoperative length of stay (median/IQR)** | 4 (3-6)  Range 0-35 | 4 (3-6)  Range 0-88 | 4 (3-6)  Range 0-23 |
| **Non-home discharge** | 120 (17.5%) | 1673 (20.9%) | 24 (14.5%) |
| **Composite (morbidity and mortality)** | 100 (14.5%) | 1250 (15.6%) | 19 (11.5%) |
| Death | 7 (1.0%) | 88 (1.1%) | 0 |
| Bleeding  Requiring Transfusion  or Secondary Procedure | 76 (11.1%) | 1010 (12.6%) | 13 (7.8%) |
| Pneumonia | 8 (1.2%) | 76 (1.0%) | 0 |
| **Arterial and venous thromboembolism (MI/stroke/VTE)** | 26 (3.8%) | 267 (3.3%) | 7 (4.2%) |
| Myocardial  Infarction or Stroke | 20 (2.8%) | 213 (2.7%) | 3 (1.8%) |
| Venous thromboembolism | 6 (0.9%) | 63 (0.8%) | 4 (2.4%) |

**Table S2: Patient characteristics of patients with missing patency data**

|  | **N (% total cohort)**  *N* = 8893 | **Missing data for patency**  *N* = 1155 (13.0%) | **Not patent**  *N* = 652 (7.3%) | **Patent**  *N* = 7086 (79.7%) |
| --- | --- | --- | --- | --- |
| **Female sex** | 2799 (31.5%) | 363 (31.4%) | 218 (33.4%) | 2218 (31.3%) |
| **Race** | | | | |
| White | 5735 (64.5%) | 727 (63.0%) | 370 (56.8%) | 4638 (65.5%) |
| Black or African American | 1354 (15.2%) | 124 (10.7%) | 124 (19.0%) | 1106 (15.6%) |
| Unknown/not reported | 1713 (19.3%) | 291 (25.2%) | 148 (22.7%) | 1274 (18.0%) |
| **ASA** | | | | |
| I | 15 (0.2%) | 1 (0.1%) | 0 | 14 (0.2%) |
| II | 397 (4.5%) | 61 (5.3%) | 15 (2.3%) | 321 (4.5%) |
| III | 6571 (73.9%) | 828 (71.7%) | 462 (70.9%) | 5281 (74.5%) |
| IV | 1897 (21.3%) | 253 (22.8%) | 172 (26.4%) | 1462 (20.6%) |
| **Symptomatology** | | | | |
| Asymptomatic | 337 (3.8%) | 56 (4.9%) | 16 (2.5%) | 265 (3.7%) |
| Claudication | 3169 (35.6%) | 408 (35.3%) | 140 (21.5%) | 2621 (37.0%) |
| Critical limb ischaemia: rest pain | 2387 (26.8%) | 306 (26.5%) | 217 (33.3%) | 1864 (26.3%) |
| Critical limb ischaemia: tissue loss | 2897 (32.6%) | 363 (31.4%) | 271 (41.6%) | 2263 (31.9%) |
| **High Risk Factors, Physiologic** | 1544 (17.4%) | 206 (17.8%) | 154 (23.6%) | 1184 (16.7%) |
| **High Risk Factors, Anatomic** | | | | |
| Prior ipsilateral bypass involving currently treated segment | 1689 (19.0%) | 225 (19.5%) | 158 (24.2%) | 1306 (18.4%) |
| Prior ipsilateral percutaneous intervention involving currently treated segment | 1551 (17.4%) | 176 (15.2%) | 116 (17.8%) | 1250 (17.8%) |
| **Readmission** | 1235 (13.9%) | 130 (11.3%) | 241 (37.0%) | 864 (12.2%) |
